# Drug Recommendation from Diagnosis Codes: Classification vs. Collaborative Filtering Approaches

**DOI:** 10.3390/ijerph20010309

**Published:** 2022-12-25

**Authors:** Apichat Sae-Ang, Sawrawit Chairat, Natchada Tansuebchueasai, Orapan Fumaneeshoat, Thammasin Ingviya, Sitthichok Chaichulee

**Affiliations:** 1 College of Digital Science, Prince of Songkla University, Songkhla 90110, Thailand; 2 Department of Biomedical Sciences and Biomedical Engineering, Faculty of Medicine, Prince of Songkla University, Songkhla 90110, Thailand; 3 Department of Ophthalmology, Faculty of Medicine, Prince of Songkla University, Songkhla 90110, Thailand; 4 Department of Family and Preventive Medicine, Faculty of Medicine, Prince of Songkla University, Songkhla 90110, Thailand; 5 Research Center for Medical Data Analytics, Faculty of Medicine, Prince of Songkla University, Songkhla 90110, Thailand

**Keywords:** machine learning, collaborative filtering, classificaiton, diseases, electronic medical prescriptions, recommender systems

## Abstract

Over time, large amounts of clinical data have accumulated in electronic health records (EHRs), making it difficult for healthcare professionals to navigate and make patient-centered decisions. This underscores the need for healthcare recommendation systems that help medical professionals make faster and more accurate decisions. This study addresses drug recommendation systems that generate an appropriate list of drugs that match patients’ diagnoses. Currently, recommendations are manually prepared by physicians, but this is difficult for patients with multiple comorbidities. We explored approaches to drug recommendations based on elderly patients with diabetes, hypertension, and cardiovascular disease who visited primary-care clinics and often had multiple conditions. We examined both collaborative filtering approaches and traditional machine-learning classifiers. The hybrid model between the two yielded a recall at 5 of 76.61%, a precision at 5 of 46.20%, a macro-averaged area under the curve of 74.52%, and an average physician agreement of 47.50%. Although collaborative filtering is widely used in recommendation systems, our results showed that it consistently underperformed traditional classification. Collaborative filtering was sensitive to class imbalances and favored the more popular classes. This study highlighted challenges that need to be addressed when developing recommendation systems in EHRs.

## 1. Introduction

Noncommunicable diseases (NCDs) are a group of diseases that are not caused by infection and cannot be transmitted through contact or a carrier [1]. They are caused by a combination of genetic, environmental, physiological, and behavioral factors. NCDs usually have a long duration and slow progression, and symptoms accumulate steadily over time. These include diabetes mellitus, hypertension, dyslipidemia, cerebrovascular diseases, heart diseases, chronic lower respiratory diseases, and cancer. NCDs account for more than 71% of annual deaths and are the leading cause of death worldwide. Nearly three quarters of all NCD deaths occur in low- and middle-income countries [2]. The increase in NCDs is largely attributable to four major risk factors: tobacco use, physical inactivity, harmful alcohol consumption, and unhealthy diets. Many people can have multiple NCDs, especially the elderly [1].

Cardiovascular disease is the leading cause of mortality in patients with diabetes, and many factors, including hypertension, contribute to this high prevalence of cardiovascular disease [3]. Hypertension is about twice as common in patients with diabetes as in those without the disease. Diabetes, hypertension, and cardiovascular disease are the most common chronic NCDs causing high mortality and morbidity worldwide. With the rapid increase in NCD-related deaths in Asia-Pacific countries, NCDs are now the leading cause of disease burden in the region [4].

The management of patients with complex comorbidities has long been considered a challenging task. For patients with diabetes, hypertension, and cardiovascular disease, a physician often prescribes drugs for one to six months, depending on their symptoms and the severity of the disease. The physician also schedules an appointment for the patient’s next visit to continue treatment. The physician may also order a pathological examination for the next visit. Together with the results of the physical examination, the results of the pathological examination are used to consider an appropriate medical prescription. Treatment of patients with diabetes, hypertension, and cardiovascular disease requires the continuous prescription of drugs. There may be increases and decreases in the amount taken, but patients must continue to take the drugs until the results of the pathological examination are satisfactory. The drugs prescribed to the patient must be appropriate for the patient’s pathological results, and the number and dosage must be correct. In this way, the drugs can be used safely by the patients.

Electronic health records (EHRs) document patients’ complete medical histories, including their diagnoses, procedures, drugs, imaging, and laboratory results. In recent decades, healthcare digitalization has increased the availability of patient data for health data analytics. The increasing adoption and use of EHRs has created a tremendous opportunity to leverage health data for clinical decision making.

Modern EHRs code patients’ diseases and conditions using diagnosis codes. The International Classification of Diseases (ICD) is a standardized classification system for diseases and conditions commonly used in EHRs globally. The ICD codes have long been used for clinical, health management, and epidemiological purposes. The ICD-10 version includes nearly 70,000 codes. Drugs can be referred to by their generic name or their brand name. The generic name does not refer to the brand of a particular company. It provides a clear and unique identification and appears on all drug and medicine labels. Generic names are often an abbreviation of the chemical name, structure, or formula of the drug. Standard terminologies, such as ICD-10 and generic names, facilitate the secondary use of EHR data and support data-driven clinical and translational research.

EHR research ranges from disease classification to readmission prediction to mortality assessment. Drug recommendation systems are one area being studied in this field. The goal of drug recommendation systems is to provide a list of relevant drugs based on the patient’s disease conditions. In recent years, recommendation systems have evolved and become indispensable for certain businesses, such as Google. Advances in recommender systems may help physicians prescribe drugs for patients by utilizing extensive EHR data. In addition to recommending a list of drugs, it is important to provide explanations for these recommendations in order to increase physicians’ acceptance of the system.

The development of recommendation systems in the medical domain has presented some challenges [5]. First, clinical data are based on variables, such as clinical measurements, medical examinations, and professional expertise, which are subjective and may be influenced by patient and physician preferences. Second, the absence of a diagnosis may mean that a person has not yet been diagnosed with a disease. However, this does not always mean that the person does not have the disease. Lastly, clinical data may consist of a variety of variable types (such as binary, continuous, categorical, and ordinal), making it difficult to model and to merge information from different sources.

Many existing drug recommendation systems have been developed based on different approaches and algorithms. For ontology- and rule-based systems, drug recommendation systems are implemented primarily based on hard-coded protocols, which are typically established by physicians and their institutions’ policies. GelenOWL [6] recommended drugs for patients using a medical and rule-based reasoning approach developed based on the patient’s disease, allergies, and known drug interactions for the drugs in the database. SemMed [7] used an ontology-based approach based on diseases, drugs, and allergies to provide a list of recommended drugs. These algorithms required the development of extensive rules, which is difficult and time-consuming to perform on a large scale.

For machine-learning-based systems, Bajor and Lasko [8] developed a deep-learning model for drug prediction which uses the most recent 100 billing codes and generates a list of suggested drugs based on their therapeutic class. LEAP [9] was a drug recommendation system for patients with complex multimorbidity which broke down the treatment recommendation into a series of decision-making steps and automatically selected the best drugs. The algorithms take the diagnosis codes of a given visit as inputs and generate a list of recommended drugs that can avoid adverse drug interactions. Wang et al. [10] developed a drug recommendation model which jointly learns the representations of patients, diseases and drugs and fuses them with a trilinear method which takes disease ontology into account. SMR [11] was a drug recommendation system based on a high-quality heterogeneous graph by bridging EMRs and medical knowledge graphs. The algorithm used heterogeneous graphs and joint-learning-embedding models to generate a list of drugs while taking into account the patient’s diagnoses and adverse drug reactions. Recently, several studies have examined specific problems in recommending drugs with more sophisticated methods. Shang et al. [12] investigated a method for drug recommendation that leverages both patient representation and drug interactions through recurrent neural networks which also take into account the patient’s previous visits. The method can effectively reduce the rate of drug-to-drug interactions in recommended medication combinations. Yang et al. [13] developed a safe drug recommendation engine based on diagnosis codes, procedure codes, drug molecule structures, and drug–drug interactions using neural networks and graph representation, which showed some improvement over using only diagnosis and procedure codes. Wu et al. [14] explored approaches which address newly approved drugs which do not have much historical prescription data using a few-shot learning problem which leverages the drug ontology to link new drugs to existing drugs with similar treatment effects and learns ontology-based drug representations. Most studies were conducted with the public MIMIC datasets [15]. Few studies have been conducted with institutional datasets. Standards for diagnosis codes, billing codes, and drug codes vary between countries. Most studies of drug recommendations relied on traditional classification methods.

Collaborative filtering, one of the most popular techniques in recommender systems, has recently been employed for clinical prediction [5]. It is based on the notion that individuals with similar preferences who agree on certain items are likely to agree on other items of which they may not be aware. Collaborative filtering can be used to generate a personalized ranking of items that are of interest. Many collaborative-filtering applications in the medical domain are for predicting comorbidities based on a patient’s clinical data, such as clinical variables or diagnosis codes. Davis et al. [16] developed a collaborative filtering model which uses patients’ ICD-9-CM codes to predict future disease risk. The model predicted each patient’s disease risk based on the patient’s own medical history and the histories of patients with similar characteristics. The output was a list of diseases for each patient, personalized and ranked by severity. Folio et al. [17] developed a similar approach for predicting comorbidities based on ICD-9-CM codes with additional layers which relied on clustering and association rules. Hassan and Syed [18] proposed a collaborative filtering model which incorporated extensive clinical variables (such as clinical measurements, diagnoses, and family history) to predict patient outcomes, i.e., sudden cardiac death and recurrent myocardial infarction. Collaborative filtering was shown to outperform traditional logistic regression and support vector machines on the same dataset. Recently, Granda Morales et al. [19] developed a drug recommendation specifically for patients with diabetes based on a user-based collaborative filtering approach which supports 10 diabetes drugs. The performance of collaborative filtering depended heavily on the data [5].

This study was motivated by the growing volume of medical records and the desire to use these data to support clinical decision making. Drug recommendation systems can assist both physicians in prescribing drugs and pharmacists in reviewing prescribed drugs. We took steps to evaluate collaborative filtering and classification approaches in the context of drug recommendation. We hypothesized that collaborative filtering could generate a list of recommended drugs based on patient characteristics, while leveraging patient similarities. This could circumvent the problem of missing data (e.g., the absence of diagnoses) in the medical domain. In this study, we focused on elderly patients with diabetes, hypertension, and cardiovascular disease because they share common risk factors and are one of the largest groups of people who frequently visit hospitals. The results of this research can be developed into a system which supports clinical decision making in drug prescription and drug verification.

## 2. Materials and Methods

Drug recommendation systems based on both classification and collaborative filtering algorithms were developed and evaluated using data from the Songklanagarind Hospital Information System. This section describes data collection, data preparation, algorithms, and evaluation procedures.

### 2.1. Dataset

Our study used retrospective cross-sectional data taken from the Hospital Information System of Songklanagarind Hospital, Thailand. We used data from patients aged more than 65 years who visited the primary-care clinics and general-practice clinics of Songklanagarind Hospital between January 2015 and December 2021 and were diagnosed with diabetes (ICD-10 code: E10-E14), hypertension (ICD-10 code: I10), or cardiovascular disease (ICD-10 code: E78). Our dataset contains patient demographics (age and sex), diagnosis codes (ICD-10), and prescribed drugs (generic name) for each outpatient visit. We did not include personal identifiable information (PII). We excluded patients who were not prescribed drugs or did not have diagnosis codes reported. The study protocol was approved by the Office of Human Research Ethics Committee, Faculty of Medicine, Prince of Songkla University under Approval No. REC.65-340-38-2.

Table 1 shows the descriptive statistics for our dataset. Our dataset consists of 28,728 outpatient visits from 3925 different patients, with an average number of visits of 7.25 per patient over five years. The total number of diagnosis codes assigned for all visits was 109,625 with 946 unique ICD-10 codes and an average of 3.82 ICD-10 codes per visit. The total number of drugs prescribed for all visits was 182,743, with 523 unique drugs and an average of 6.36 drugs per visit. Data entry standards were consistent across the dataset.

**Figure 1:**
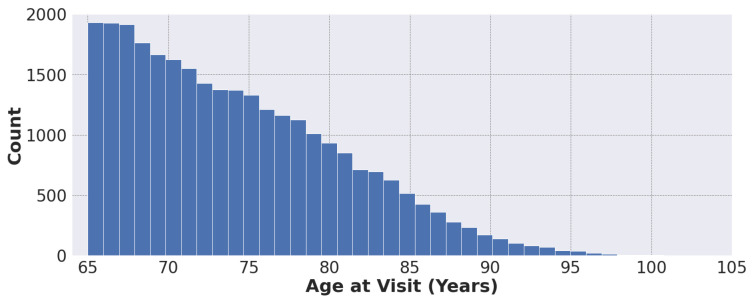
Distribution of patient age at visit (N = 28,728).

Figure 1 shows the distribution of patient age at visit in our dataset, ranging from 65 to 105 years.

### 2.2. Data Preparation

Patients come to the doctor for a wide variety of reasons. Our dataset contains 946 unique ICD-10 codes and 523 unique generic codes for drugs (see Table 1). Some diseases and conditions have a high prevalence, while others are coded less frequently. The same applies for drugs. It is difficult to develop a model which supports all ICD-10 codes and all drugs. Some less frequently coded features may not have strong predictive power or may not be sufficient to represent the problem.

**Figure 2:**
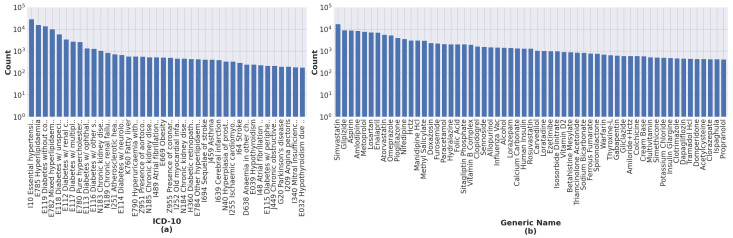
Distribution of (**a**) ICD-10 codes and (**b**) drugs’ generic names for the 40 most frequent ICD-10 codes and the 60 most frequent generic names of drugs in the dataset. All chosen features have at least 200 occurrences.

**Figure 3:**
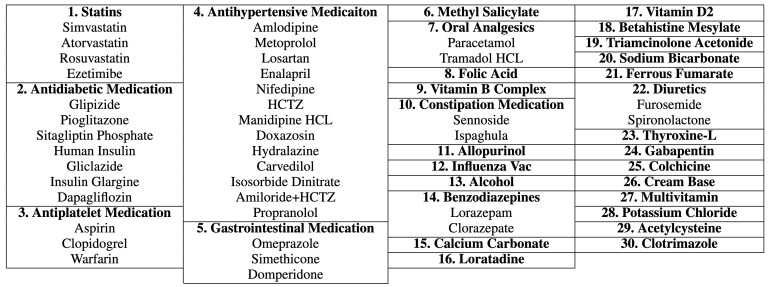
Medications are grouped according to their therapeutic function. Different drugs may belong to the same group.

**Figure 4:**
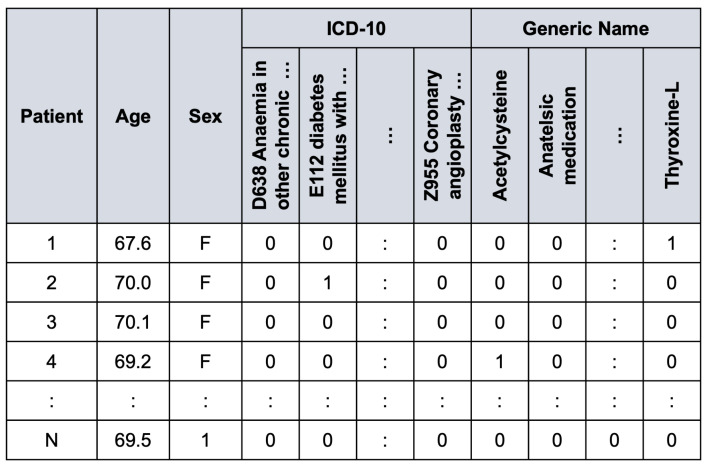
Example of the preprocessed data frame.

Our process of data transformation was as follows. First, we selected the 40 most frequent ICD-10 codes and the 60 most frequent generic names of drugs. All selected features have more than 200 occurrences. Figure 2 shows the distribution of ICD-10 codes and drugs’ generic names for all records. Second, because different drugs have similar therapeutic functions, we grouped drugs according to their therapeutic functions using a panel of physicians. This resulted in 30 different drug groups from 60 individual generic names of drugs, as shown in Figure 3. Finally, the categorical variables, i.e., ICD-10 codes and generic names of drugs, were transformed into dummy or indicator variables which take the value 0 or 1 only to indicate the presence or absence of each category. Figure 4 illustrates an example of the preprocessed data frame used in the study.

We randomly divided our preprocessed data into two sets: a training set (90%); and a test set (10%). Patients in the training and test sets were assumed to be mutually exclusive. This resulted in the training set containing 25,855 inpatient visits, whereas the test set contained 2873 inpatient visits. We used 10-fold cross-validation in the training set to find optimal hyper-parameters and select the best model to evaluate with the test set.

### 2.3. Classification Algorithms

Classification is a predictive modeling problem which predicts a class label for input data. A model uses the training dataset with many examples of inputs and outputs to learn to map input data to class labels. Drug recommendation can be formulated as a supervised multi-class multi-label classification problem in which patient demographics and diagnosis codes are the inputs and the drugs are outputs. In this study, four different classification algorithms were assessed: nearest neighbors, logistic regression, random forest, and multilayer perceptron.

**Nearest neighbors** [20] looks for a certain number of training samples which are closest to the new data and then uses them to predict the class label by a simple majority vote of the closest neighbors of the new data. Probability scores are a fraction of votes among the closest neighbors. Nearest neighbors was a simple baseline method for our classification models. We implemented a nearest neighbor algorithm with 24 nearest neighbors and the Minkowski distance metric.

**Logistic regression** [20] is a statistical model which predicts the probability of an event based on independent variables. Given a set of *m* input variables $x=\{x_{1},x_{2},\ldots ,x_{m}\}$, the binary logistic function has the form:

(1) $$p\left(x\right)=\frac{1}{1+e^{-(\beta_{0}+\beta_{1}x_{1}+\beta_{2}x_{2}+\cdots +\beta_{m}x_{m})}}$$

where $\beta =\{\beta_{0},\beta_{1},\beta_{2},\ldots ,\beta_{m}\}$ are the regression coefficients learned from the data by minimizing a loss function. We used the lbfgs optimizer [21] with L2 regularization and a C parameter of 1.

**Random forests** [22] is an ensemble learning model based on multiple decision trees created from the training data. Decision trees are a popular method for non-parametric supervised-learning problems. However, deep decision trees tend to have low bias but high variance because they often overfit their training data. The strategy of random forests is to average multiple shallow decision trees trained from different parts of the same training set with the aim of reducing variance. The output of random forests is the class label chosen by the majority of the decision trees. Probability scores are aggregated by averaging the class probability estimates for all decision trees. In our implementation, we used the Gini impurity criterion to estimate the best feature for the split, the number of decision trees of 200, and the maximum tree depth of 8.

**Multilayer perceptron** [20] is a fully connected feedforward artificial neural network consisting of an input layer, one or more hidden layers, and an output layer. One layer can have multiple nodes. Each node is equipped with a weighted neuron with a nonlinear activation function. Every node in one layer has a weighted connection to every node in the following layer. Learning is performed by adjusting the weights of each neuron based on the errors in comparison to the expected results. The inputs pass through each layer in turn and are weighted until they reach the output layers. Subsequently, the softmax function is applied to normalize the output of the network into a vector of probability scores. Multilayer perceptron is able to find approximate solutions to complex problems. We implemented a multilayer perceptron network with two hidden layers in which each layer has 128 nodes. Each node was equipped with a ReLU activation function. The network was trained using the Adam optimizer with a constant learning rate of 0.001 until the loss did not improve by 0.0001 over 10 consecutive iterations.

We used the scikit-learn library [23] on Python 3.8.10 for the development of all classification algorithms. In our case, with multiple classes and multiple labels, the one-vs-rest scheme was used to train multiple binary classification models in which each model is responsible for only one class label. All hyperparameters, as listed above, were determined by 10-fold cross-validation on the training set. We provided class weights which are inversely proportional to class frequencies to compensate for imbalances between classes.

### 2.4. Collaborative Filtering Algorithms

Relational learning is the process of determining unknown values in a relationship utilizing a database of entities and their relationships to each other [24]. In our case, we employ relational learning for drug recommendation, where the entities include patients, drugs, and diagnoses. The relationships encode the drugs prescribed by physicians and the diagnoses of the patients. In domains with multiple relationships, information from one relationship can be used to predict another.

Relational data is composed of entities and the interactions between them [24]. In many relational domains, the number of entity types and interactions is often fixed. They may consist of only one or two entity types, such as patients and drugs. Given a relational schema with *t* entity types: $\varepsilon_{1},\ldots ,\varepsilon_{t}$ in which $\epsilon_{t}\in \varepsilon_{t}$. An interaction between a pair of entity types, $\varepsilon_{i}$ and $\varepsilon_{j}$, is denoted as an $n_{i}\times n_{j}$ interaction matrix, $X^{\left(ij\right)}$, where $n_{i}$ is the number of $\epsilon_{i}$ entities and $n_{j}$ is the number of $\epsilon_{j}$ entities. The element $x_{\epsilon_{i},\epsilon_{j}}\in X^{\left(ij\right)}$ specifies an interaction between two entities, $\epsilon_{i}$ and $\epsilon_{j}$. Using the low-rank matrix factorization, the interaction matrix $X^{\left(ij\right)}$ can be expressed as the product of two lower dimensional matrices:

(2) $$X^{\left(ij\right)}\approx f^{\left(ij\right)}\left(U^{\left(i\right)}{\left(U^{\left(j\right)}\right)}^{T}\right)$$

where $U^{\left(i\right)}\in R^{n_{i}\times k_{ij}}$, $U^{\left(j\right)}\in R^{n_{j}\times k_{ij}}$ and $k_{ij}\ll n_{i},n_{i}$. The two lower dimensional matrices, $U^{\left(i\right)}$ and $U^{\left(j\right)}$, can be thought of as latent factors determined for entity type $\varepsilon_{i}$ and $\varepsilon_{j}$, respectively. The latent dimension *k* is the number of latent factors and $f^{\left(ij\right)}$ is a non-linear indicator function [24]. If $\varepsilon_{i}$ is involved in more than one interaction, each interaction can be modeled separately. The latent factors, $U^{\left(i\right)}$ and $U^{\left(j\right)}$, can be obtained by minimizing a loss function [25] based on the observed interaction matrix, $X^{\left(ij\right)}$:

(3) $$\min_{U^{\left(i\right)},U^{\left(j\right)}}\left|\right|f^{\left(ij\right)}\left(X^{\left(ij\right)}-b^{\left(i\right)}-b^{\left(j\right)}-U^{\left(i\right)}{\left(U^{\left(j\right)}\right)}^{T}\right)\left|\right|$$

where $b^{\left(i\right)}$ and $b^{\left(j\right)}$ are biases for $\varepsilon_{i}$ and $\varepsilon_{j}$, respectively [25]. The prediction for the unknown values of $X^{\left(ij\right)}$ can be obtained by the product of the two latent factors, $U^{\left(i\right)}$ and $U^{\left(j\right)}$. Recommendations can be made by sorting the predictions in descending order.

**Collective matrix factorization** (CMF) extends the low-rank matrix factorization by jointly factorizing the interaction matrices along with their side information, while sharing the latent factors between them [25]. Our CMF model was based on two entity types: $\varepsilon_{1}$ for patients and $\varepsilon_{2}$ for drugs. The CMF model jointly factorizes the interaction matrix $X^{\left(12\right)}$, which indicates which drug is prescribed for which patient, along with the patient attribute matrix $S^{\left(1\right)}$, which indicates patient demographics and the diagnoses a physician makes for each patient, and the drug attribute matrix $S^{\left(2\right)}$, which indicates the distribution of each diagnosis code for each drug. Using low-rank matrix factorization, the side attribution matrices can be expressed as $S^{\left(1\right)}\approx U^{\left(1\right)}{\left(V^{\left(1\right)}\right)}^{T}$ and $S^{\left(2\right)}\approx U^{\left(2\right)}{\left(V^{\left(2\right)}\right)}^{T}$, where $V^{\left(1\right)}$ and $V^{\left(2\right)}$ are the two new latent factors for the patient and drug attribute matrices, respectively. The latent factors can be obtained by minimizing a squared loss:

(4) $$\min_{U^{\left(1\right)},U^{\left(2\right)},V^{\left(1\right)},V^{\left(2\right)}}\left|\right|f^{\left(12\right)}\left(X^{\left(12\right)}-b^{\left(1\right)}-b^{\left(2\right)}-U^{\left(1\right)}{\left(U^{\left(2\right)}\right)}^{T}\right){\left|\right|}^{2}\;+\left|\right|S^{\left(1\right)}-U^{\left(1\right)}{\left(V^{\left(1\right)}\right)}^{T}{\left|\right|}^{2}\;+\left|\right|S^{\left(2\right)}-U^{\left(2\right)}{\left(V^{\left(2\right)}\right)}^{T}{\left|\right|}^{2}$$

where $U^{\left(1\right)}$ and $U^{\left(2\right)}$ are shared between factorizations [25]. This non-convex optimization can be solved using the alternating least squares method to find local minima.

**Offsets** [25] is a recommendation model which provides another alternative to the CMF model in which the low-rank matrix is decomposed into linear additive components:

(5) $$X^{\left(ij\right)}\approx f^{\left(ij\right)}\left(\left(U^{\left(i\right)}+S^{\left(i\right)}V^{\left(i\right)}\right){\left(U^{\left(j\right)}+S^{\left(j\right)}V^{\left(j\right)}\right)}^{T}\right).$$

The latent vectors are obtained by optimizing a squared loss based on the observed interaction matrix:

(6) $$\min_{U^{\left(1\right)},U^{\left(2\right)},V^{\left(1\right)},V^{\left(2\right)}}\left|\right|f^{\left(12\right)}\left(X^{\left(12\right)}-b^{\left(1\right)}-b^{\left(2\right)}-\left(U^{\left(1\right)}+S^{\left(1\right)}V^{\left(1\right)}\right){\left(U^{\left(2\right)}+S^{\left(2\right)}V^{\left(2\right)}\right)}^{T}\right){\left|\right|}^{2}.$$

The Offsets model is aimed at making cold-start recommendations because the predictions can be obtained by a simple vector–matrix product rather than by solving a complex linear system.

**Most Popular** [25] is a recommendation model which fits a model with only biases in order to provide non-personalized recommendations (see Equation (3)). The Most Popular model is a simple model which resembles the CMF model without the latent factors that serve as a benchmark.

In our study, we applied the CMF, Offsets, and Most Popular models to our dataset. Similar to classification approaches, 10-fold cross-validation was applied to the training set to find appropriate model hyperparameters. Our models were then evaluated with the test set.

### 2.5. Hybrid Strategy

The prediction scores from both classification and collaborative filtering models, as computed above, can be combined into a final prediction score as follows:

(7) $$p_{\mathrm{combine}}\left(x\right)=\alpha \cdot p_{\mathrm{class}}\left(x\right)+\left(1-\alpha \right)\cdot p_{\mathrm{collab}}\left(x\right)$$

where α is a weighting factor. The appropriate α value was determined by cross-validation. The class labels which had scores in the top *K*, or more than a predefined threshold, were recommended.

### 2.6. Evaluation Metrics

We performed inference for each model on the hold-out test set to obtain a top-*K* of recommended drugs, ranked by their scores, for each outpatient visit. Results were compared with a list of actual prescribed drugs. We employed the following evaluation metrics:

- Recall at *K* (Recall@*K*) is the proportion of the actual drugs prescribed for a given patient that are included in the top-*K* recommendation list:
  (8) $$\mathrm{Recall}@K=\frac{1}{\left|\tau \right|}\sum_{i=1}^{K}\left\{\begin{array}{c} \;1\;\;\;\;\;\;\;\;\;\;\;\;r_{i}\in \tau \\ 0\;\;\;\;\;\;\mathrm{otherwise} \end{array}\right.,$$
  where $r_{i}$ is the recommended item ranked at position *i* (sorting by the prediction scores in descending order) and τ is the set of actual drugs prescribed by a physician).
- Precision at *K* (Precision@*K*) is the proportion of top-*K* recommendation list that contains the actual drugs prescribed for a given patient:
  (9) $$\mathrm{Precision}@K=\frac{1}{k}\sum_{i=1}^{K}\left\{\begin{array}{c} \;1\;\;\;\;\;\;\;\;\;\;\;\;r_{i}\in \tau \\ 0\;\;\;\;\;\;\mathrm{otherwise} \end{array}\right..$$
  Precision@*K* is the most intuitive metric, which captures what a recommendation system is aiming for.
- Hit rate at K (Hit@*K*) is a metric which examines whether any of the top-*K* recommended items were included in the list of actual drugs prescribed for a given patient.
- Normalised discounted cumulative gain at *K* (NDCG@*K*) is a metric which considers the presence of an item in the top-*K* recommendation list and applies a discount according to the rank of each item in the top-*K* list.
- Average precision (AP) calculates the average value of precision over precision-recall pairs for different thresholds. It describes how well a system recalls the actual items in the top ranks.
- Area under the receiver-operating characteristic curve (AUROC) takes the entire ranking of items and gives a standardized number between 0 and 1. AUROC describes the overall classification ability of the system.

In our evaluation scheme, we used K=5 and calculated the macro average and macro average across class labels for both AP and AUROC.

### 2.7. Physician Evaluation

While it was tempting to test the model under real-world conditions, this required a certain level of confidence in the model and passing several rounds of the assurance process. Instead, we developed a web-based system to ask whether a physician would accept or reject the list of recommendations if it appeared when prescribing medication.

From the test set, 200 inpatient visits were selected to create a physician evaluation set using stratified random sampling, with each ICD code associated with at least ten inpatient visits. This was done to ensure that our physician evaluation set covered all ICD codes and drugs.

For each inpatient visit, our highest-scoring model processed patient demographics and ICD codes to suggest a list of drugs whose scores were above the threshold, which yielded a recall score of 0.80 in the validation set. We presented the input and the output to three physicians and asked whether they agreed or disagreed with a list of recommended drugs. We asked the physicians to make a judgment based on diversity, explainability, and ranking. After all physicians completed the task, we calculated the percentage of when physicians agreed with the recommended drugs and a Fleiss’ kappa coefficient which describes agreement between annotators.

## 3. Results

This section presents the performance of the classification, collaborative filtering, and hybrid models. For each model, we performed 10-fold cross-validation to optimize and select the best hyperparameters. During the cross-validation, we found neither under-fitting nor over-fitting for all models. All models were evaluated with the hold-out test set. The list of recommended drugs was compared with the actual prescribed drugs using the evaluation metrics mentioned in the previous section, and it was assessed by physicians whether they agreed with the recommendations.

### 3.1. Recommendation Performance

Table 2 shows the performance of the classification, collaborative filtering, and hybrid models for recommending drugs on the hold-out test set. Among the classification algorithms, the multilayer preceptron model, which was based on feedforward neural networks, scored highest on most metrics. Among the collaborative filtering algorithms, the Offsets model, which was based on linear additive combinations, scored highest on most metrics. Overall, the classification models outperformed the collaborative filtering models. The hybrid model, which was based on weighting the scores from the best classification and collaborative filtering models, scored highest on most metrics.

Table 3 details the AUCs of the multilayer preceptron, Offsets, and hybrid models when recommending each drug in the test set. The Offsets model performed worse compared with the other models for all drugs. The hybrid model performed better on the more common drugs. The differences in performance between the multilayer perceptron model and the Offsets model were much more pronounced for less common drugs.

**Figure 5:**
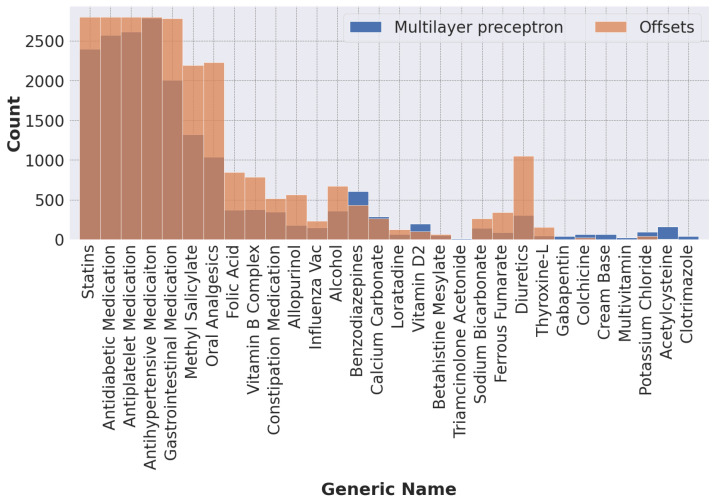
Distribution of recommended drugs in the test set.

Figure 5 shows the distribution of recommended drugs at the threshold, which yielded a recall score of 0.80 in the validation set. The multilayer perceptron model offered clear advantages over the Offsets model, which did not perform well for the less common drugs. It is more biased toward popular drugs.

### 3.2. Physician Evaluation

**Figure 6:**
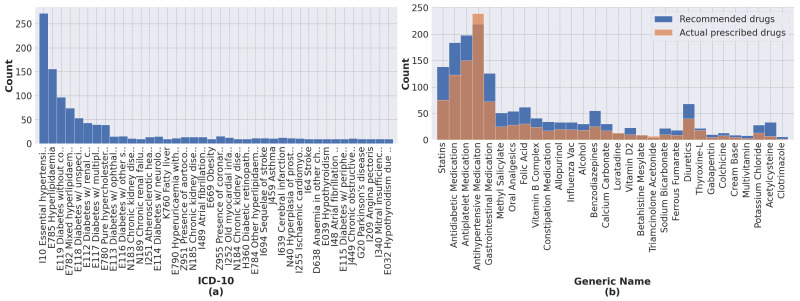
Distribution of (**a**) ICD-10 codes and (**b**) recommended drugs in our physician evaluation set.

Figure 6 shows the histogram of ICD codes, actual prescribed drugs, and recommended drugs in our physician evaluation set of 200 inpatient visits. The average number of ICD codes, actual prescribed drugs, and recommended drugs were 5.38, 4.86, and 7.35 per inpatient visit, respectively.

We ran each sample in our physician evaluation set through the hybrid model to obtain a list of recommended drugs for physician evaluation, as the hybrid model scored highest overall. The average of the percentages upon which physicians agreed with the recommendations was 47.50% with a multi-rater Fleiss’ Kappa coefficient of 30.54% (see Table 4).

## 4. Discussion

The growing amount of medical records has motivated the secondary use of data to support clinical workflow. Drug recommendation systems learn from the diagnostic and prescription data already in the system to suggest drugs which might be of interest to a physician. The systems recommend drugs that correspond to the patient’s diagnostic concerns. This could reduce the time it takes to prescribe drugs in the EMR system. The model trained for the drug recommendation system could also be used to automatically review prescriptions to determine if they are consistent with their diagnostic details.

The objective of the study was to examine a problem of drug recommendation using classification-based and collaborative filtering-based algorithms on real-world hospital data. In this present study, we specifically focused on patients with diabetes, hypertension and cardiovascular disease, which were prevalent in primary-care clinics. This allowed a better understanding of the performance of different recommender systems on medical data which may have different characteristics compared to other real-world data.

Although collaborative filtering is widely used to provide personalized recommendation by collecting data characteristics from many subjects, we found that collaborative filtering did not perform well in our problem. All classification approaches outperformed the best collaborative filtering model, even with the simplest nearest-neighbor classification.

For the classification models, multilayer perceptron slightly outperformed random forests, and a large difference was observed in the macro-averaged AUC. Both models have proven successful on nonlinear problems and are inherently flexible for mixed predictors of continuous, categorical, and binary variables. In our view, both models can well handle severe class imbalance and missing data, which are common in medical data.

Among the collaborative filtering models, the collaborative matrix factorization and Offsets models that account for side information performed better than the most popular model, which was the baseline. While differences between recommendation metrics for the top-five recommendations were marginal, large differences in performance were observed for AP and AUC values. When comparing the best classification model, multilayer perceptron, with the best collaborative filtering model, Offsets, a large difference in performance was observed. This may be because our dataset is largely unbalanced, which may cause collaborative filtering to give unfair predictions for minority classes. They did not seem to capture some of the less frequently coded labels (see Figure 5). This problem could be mitigated by a larger dataset.

The purpose of implementing the hybrid model was to combine the scores of the best classification and collaborative filtering models into a single score in order to improve performance. As expected, the hybrid model produced the best overall results, even with the simple weighting combinations. Unfortunately, because the Offsets model performs poorly on less frequent labels, it also degrades the performance of the hybrid model on less frequent labels (see Table 3).

Due to the inherent complexities of the drug recommendation problem, there were a number of uncertainties in both the task itself and the data. For example, the chief complaints, which are the main reason for the patient’s visit, were not explicitly coded in our dataset. We only have the patient’s demographic data and a list of unranked diagnosis codes. It is possible that relevant drugs were not prescribed because a patient already had them on hand from previous visits. It is also possible that patients came to the consultation for reasons unrelated to the chronic diseases in which we are interested. Some degree of error was, therefore, to be expected.

### 4.1. Comparison to Other Studies

Our results are consistent with those of Hao and Blair [5], who investigated classification and collaborative filtering approaches for clinical prediction on various simulated and real-world datasets. Their study focused on the performance of the algorithms under different degrees of missing data. They concluded that the collaborative filtering approach was consistently inferior to classification-based approaches, such as logistic regression and random forests, under various imputations on both real-world and simulated data. They suggested that collaborative filtering might not be desirable in the clinical setting, where classification may be an acceptable alternative. Although there was a slight difference in both the implementation of the collaborative filtering algorithms, where our algorithms take into account side information, and the objective of the algorithm, i.e., clinical prediction vs. drug recommendation. We observed similar results with collaborative filtering. Collaborative filtering performed poorly on datasets with severe class imbalances.

Our results differ from those by Hassan and Syed [18], which used collaborative filtering for clinical prediction. They reported that collaborative filtering approaches had a higher prediction accuracy than classification counterparts for certain tasks, i.e., sudden cardiac death and recurrent myocardial infraction. They noted that collaborative filtering exploits similarity between individual patients in the historical dataset in determining patient risk by comparing new patients to historical datasets. They also found that collaborative filtering can provide benefits when the data are complete, that is, without missing data or unknown outcomes.

Compared with other studies in the field of drug recommendations, many studies were based on complicated rule-based ontology reasoning approaches which consider domain knowledge, such as drug–drug interactions [9,10,11,12,13,14]. Compared with our study, which obtained a micro-averaged AUC of 90.09% for the hybrid model on the de-identified Songklanagarind’s EHR dataset, Bajor and Lasko [8] obtained a micro-averaged AUC of 92.70% on the de-identified Vanderbilt’s EHR dataset, and Wu et al. [14] obtained micro-averaged AUCs of 86.08% and 72.75% on the MIMIC-IV dataset and the Claims dataset, respectively. These results are slightly different because the studies were designed differently and different datasets were used. Our approaches were based on the knowledge discovered by learning patterns in data, similar to Bajor and Lasko’s study [8]. In their study, they trained a recurrent neural network model on a larger dataset of over 600,000 patient records. Similar to us, they grouped similar drugs based on their therapeutic class (e.g., beta blockers, diuretics, and immune suppressants), resulting in over 1000 drugs grouped into 182 therapeutic classes. Their model processed 100 recent ICD-9 codes to generate a list of suggested drugs. It was reported that their recurrent neural network model outperformed a feedforward neural network model by 1 percent in micro-averaged AUC. Such a small difference could possibly be noticeable in clinical use [8]. While we focused on the patient’s current visit, their approaches may be biased toward patients who visit the hospital frequently compared to patients who are visiting the hospital for the first time. To date, no study has examined both classification and collaborative filtering approaches together for drug recommendation.

### 4.2. Physician Acceptance

Physician acceptance is critical to getting the most value from systems designed to support physicians. They must be accurate, because physicians will ignore inaccurate and ineffective decision support systems. They must support existing clinical workflows without requiring additional inputs or actions. They must help physicians improve the quality of care while maximizing their own productivity and efficiency.

When developing a recommendation system, we can expect an algorithm to have other good algorithmic properties besides numerical metrics, such as diversity and explainability. We do not want a user to be trapped in the confinement area of popular classes. Currently, none of the studies take physician acceptance into account. We conducted a human-based evaluation to assess the quality of the recommendations provided by the systems. We obtained an average agreement rate of 47.50% with an inter-rater coefficient of 30.54% (see Table 4). This indicates that physicians agreed with the list generated from the recommendation model fairly often. Physicians criticisms included that the algorithms often recommended drugs that they did not think were relevant to the patient condition based on the given ICD-10 codes alone, e.g., analgesics and diuretics. If the algorithm was integrated with a drug–drug interaction database, a disease ontology database, and a drug ontology database, this problem could still occur. To address the problem, the algorithm should process the patient’s chief complaint or clinical notes in addition to ICD-10 codes. More studies, however, need to be carried out on the algorithms to see how the systems are ready to be used and adopted by physicians.

### 4.3. Study Limitations

The study was subject to certain limitations. First, we did not consider domain knowledge, such as disease ontology or drug–drug interactions, in developing the models. We relied solely on the model to discover complex patterns in the data. Integrating these medical concepts could improve drug recommendation and physician acceptance. Second, apart from the diagnosis codes, we did not consider any other context around the visit, such as physical examination, laboratory tests, and patient notes. Third, with collaborative filtering, it is notoriously difficult to incorporate side features for items. We used collective matrix factorization approaches [25], which can incorporate side features, but little performance improvement was observed. Deeper investigation of approaches, e.g., with advanced feature embedding, could lead to further improvements in the results. Finally, our model did not consider the patient’s historical records. Past medical records can provide information about what medications the patient was prescribed in the past and what medications the patient is currently taking. Incorporating these into the model would allow physicians to make better recommendations and lead to better physician acceptance. Models based on long short-term memory (LSTM), gated recurrent units (GRU) or transformers which can handle sequential or historical data could further improve results.

### 4.4. Future Work

Our recommendation systems can be improved by considering more comprehensive patient profiles (such as current and historical weight, height, body mass index, laboratory tests, diagnosis, and treatment), domain knowledge (such as disease ontology and drug–drug interactions), as well as laboratory and diagnostic tests when making recommendations. Drug recommendation systems can also be improved by incorporating unstructured data such as clinical notes, both from nurses and physicians, which often contain important contextual information about each patient visit. For example, patients’ chief complaints, which indicate the main reasons for their visits, are often written as free text in clinical notes. The chief complaints might include information about the patient’s condition which is sometimes not included in diagnosis codes, thus improving the outcomes of recommendations. Such an implementation requires more complex feature engineering and neural network architectures, such as a language model for processing natural language data and a recurrent neural network model for processing clinical data with irregular times. Although it seems that collaborative filtering approaches may also be appropriate for this context due to their scalability and dynamic learning, the present study and Hao and Blair’s study [5] prove otherwise. They do not handle complex clinical data well. In addition, recommendation systems can also be extended to support more disease codes and drugs by leveraging a larger set of clinical data. Further investigation into more advanced algorithms and more comprehensive clinical data is our focus for future work.

### 4.5. Implementation Considerations

The overabundance of medical information has made it difficult for health-care professionals to make patient-centered decisions. These difficulties highlight the need to implement healthcare recommendation systems to help both end users and healthcare professionals make more efficient and accurate clinical-related decisions. These systems must gain the confidence of users in the sense that they draw robust and causal inferences from clinical data. They must also be fast enough and integrate well with the current system. Such complex artificial intelligence systems may need to be deployed locally at the edge of the system to enable rapid performance. Sever-side processing may result in some latency. Deployment considerations must be made before developing clinical recommendation engines.

Recommendation systems are often based on medical codes (such as medicinal code, ICD-9, ICD-10, or the most recent ICD-11). The use of these codes varies from country to country and from institution to institution. There are also some variations of these codes, such as country-specific extensions. At some point, there may be a change from the current version to a newer version. Although mapping between different versions is possible, the newer versions of the codes generally include more diseases and symptoms and may be more specific. Some institutions may use SNOMED-CT, which contains more detailed clinical information than ICD variants. It could also be that new drugs will be introduced at some point. This creates hurdles in implementing the systems and makes it difficult to maintain them. The system must be dynamic enough to cope with such constant changes. It is certainly difficult to create a one-size-fits-all solution.

## 5. Conclusions

Recommendation systems can help healthcare professionals make better and faster clinical decisions in this age of overloaded medical information. This study aims to explore approaches for drug recommendations in patients with diabetes, hypertension and cardiovascular disease on a real-world institutional dataset. Drug recommendation systems learn from the diagnostic and prescription data already in the EHR system to recommend drugs that correspond to the patient’s diagnostic concerns to a physician. We investigated both collaborative filtering approaches and traditional machine-learning classifiers. Although collaborative filtering is widely used in recommender systems to provide personalized recommendations based on data characteristics from many subjects, collaborative filtering consistently underperformed traditional classification in our problem of interest. We observed that collaborative filtering was sensitive to severe class imbalances and tended to favor more popular labels. Performance improvements could be observed by incorporating more comprehensive patient profiles into the learning process. Drug recommenders could be an important tool for healthcare professionals as they can streamline clinical workflow. There are practical and implementation issues that need to be considered, such as deployability, prediction latency, clinical coding changes, and long-term maintainability.

## Figures and Tables

**Table 1:** Descriptive statistics of the dataset.

Characteristics	
Duration	January 2015 to December 2021
Number of visits	28,728
Number of patients	3925
Number of visits per patient ^1^	7.25 (6.46)
Age ^1^	74.25 (6.66)
Sex	
Male ^2^	1676 (42.27%)
Female ^2^	2289 (57.73%)
Number of diagnosis codes ^3^	109,625 (946)
Number of diagnosis codes per visit ^1^	3.82 (1.18)
Number of prescribed drugs ^3^	182,743 (523)
Number of prescribed drugs per visit ^1^	6.36 (2.94)

^1^ mean (standard deviation); ^2^ frequency (proportion); and ^3^ total count (unique values).

**Table 2:** Performance of the classification, collaborative filtering, and hybrid models for recommending drugs on the test set.

	Recall@5	Precision@5	Hit@5	NDCG@5	Macro AP	Micro AP	Macro AUC	Micro AUC
**Classification Models**								
Nearest neighbors	74.76	44.67	96.89	77.06	24.53	61.56	70.62	88.10
Logistic regression	74.78	44.63	97.07	77.26	22.94	63.35	69.32	88.54
Random forests	76.11	**46.31**	**97.49**	76.09	**32.95**	64.10	73.23	89.49
Multilayer perceptron	**76.19**	45.94	96.89	**78.56**	32.50	**67.40**	**75.65**	**89.67**
**Collaborative filtering models**								
Most popular	73.18	43.55	95.92	75.02	11.01	53.96	50.00	84.22
Collaborative matrix factorization	73.78	43.71	**96.78**	76.07	14.52	59.26	58.89	85.90
Offsets	**73.87**	**43.81**	96.75	**76.31**	**15.79**	**60.78**	**60.27**	**86.28**
**Hybrid model**	**76.61**	**46.20**	**97.00**	**78.97**	32.53	**68.26**	74.52	**90.09**

The bold texts indicate the algorithms that achieved the highest score for each evaluation metric.

**Table 3:** Performance of the multilayer perceptron, Offsets, and hybrid models for recommending each drug in the test set.

	N	AUC		
		Multilayer Perceptron	Offsets	Hybrid
1. Statins	726	70.17	59.91	**70.33**
2. Antidiabetic Medication	1331	69.55	60.50	**69.73**
3. Antiplatelet Medication	1041	**75.61**	65.43	75.52
4. Antihypertensive Medicaiton	2478	**71.92**	62.78	71.91
5. Gastrointestinal Medication	541	70.50	62.17	**70.64**
6. Methyl Salicylate	288	**66.26**	51.28	66.00
7. Oral Analgesics	231	**67.86**	51.52	67.69
8. Folic Acid	201	74.34	64.08	**75.00**
9. Vitamin B Complex	190	72.77	57.71	**73.24**
10. Constipation Medication	127	69.64	61.91	**70.89**
11. Allopurinol	120	85.15	75.11	**86.15**
12. Influenza Vac	151	61.05	58.71	**61.53**
13. Alcohol	156	78.10	69.69	**79.12**
14. Benzodiazepines	190	71.43	56.96	**71.58**
15. Calcium Carbonate	122	80.45	64.55	**80.83**
16. Loratadine	105	**72.25**	51.82	71.32
17. Vitamin D2	90	**83.80**	61.74	83.51
18. Betahistine Mesylate	104	**63.79**	41.70	61.82
19. Triamcinolone Acetonide	86	**68.05**	48.99	67.19
20. Sodium Bicarbonate	65	90.83	84.55	**92.87**
21. Ferrous Fumarate	72	81.82	70.66	**82.32**
22. Diuretics	204	87.66	78.91	**87.68**
23. Thyroxine-L	66	**82.25**	62.61	80.78
24. Gabapentin	55	**69.17**	56.15	68.88
25. Colchicine	44	**78.25**	47.32	74.25
26. Cream Base	55	**77.48**	62.99	77.04
27. Multivitamin	58	67.24	46.60	**67.77**
28. Potassium Chloride	55	**85.28**	60.83	83.80
29. Acetylcysteine	42	**78.67**	63.70	78.63
30. Clotrimazole	42	67.44	47.18	**67.66**

The bold texts indicate the algorithms that achieved the highest score for each drug.

**Table 4:** Physician acceptance of drug recommendations.

Acceptance (%)				Inter-Rater Agreement
Physician A	Physician B	Physician C	Mean	
34.50	52.00	56.00	47.50	30.54

## Data Availability

The data presented in this study are available on request from the corresponding author. The data are not publicly available due to the institutional policy.
